# Electronic nose versus VITEK 2 system for the rapid diagnosis of bloodstream infections

**DOI:** 10.1007/s42770-023-01154-4

**Published:** 2023-10-23

**Authors:** Ehab I. Mohamed, Mohamed S. Turkey, Radwa A. Meshref, Abeer A. Ghazal, Sherine M. Shawky, Aliaa G. Aboulela

**Affiliations:** 1 Medical Biophysics Department, Medical Research Institute, Alexandria University, Alexandria, Egypt; 2 Microbiology Department, Medical Research Institute, Alexandria University, Alexandria, Egypt; 3 Microbiology and Immunology Department, Faculty of Pharmacy, October 6 University, Sixth of October City, Giza, Egypt; 4 Medical Equipment Technology Department, Faculty of Applied Health Sciences Technology, Pharos University, Alexandria, Egypt

**Keywords:** Electronic nose (E-Nose), BACTEC, VITEK 2, *E. coli*, *K. pneumonia*

## Abstract

Infectious diseases that spread through the bloodstream, known as bloodstream infections (BSIs), are a major global health problem. Positive outcomes for patients with sepsis are typically the result of prompt treatment started after an early diagnosis of BSIs. In this study, we evaluated the capabilities of a portable electronic nose (E-Nose) to detect BSIs with two commonly isolated Gram-negative bacterial species, *E. coli* and *K. pneumonia*. One hundred and five blood samples were randomly collected for blood culture examinations using BACTEC and VITEK 2 system, and headspace analysis by an E-Nose from June to December 2021. Classification accuracy of *E. coli*, *K. pneumonia*, and negative controls was measured using principal component analysis, area under the receiver operating characteristic curve, sensitivity, and specificity analysis. After incubation for 24 h, cluster plots generated using principal component analysis demonstrated that E-Nose could accurately diagnose the presence of *E. coli* and *K. pneumonia* in BACTEC blood culture bottles with a sensitivity and specificity of 100% in just 120 s. The E-Nose method has been shown to be an immediate, precise, and cost-effective alternative to automated blood culture BACTEC and VITEK 2 systems for the fast detection of the causative bacterial pathogens of BSIs in clinical practice. Thus, patients with such Gram-negative bacteremia can have guided empirical antimicrobial therapy on the same day of BSIs diagnosis, which can be lifesaving.

## Introduction

Patients with bloodstream infections (BSIs; a term for the presence of bacteria, fungi, or viruses in a blood culture) are at an increased risk for complications and death [1, 2]. Although blood cultures of venous blood samples are widely used to diagnose bacterial infections, they have significant limitations, including high turnaround times, low sensitivity, and low specificity [3]. Over the last two decades, significant improvements have been made in diagnosing BSIs by automating blood culture procedures via VITEK systems [4, 5]. However, high costs, time constraints, labor participation, and contamination continue to be barriers to conventional and automated infectious disease diagnostic procedures [6]. As a result, faster and more precise pathogen identification procedures are needed to begin targeted therapy as soon as possible.

Recent advances in complex chemical detection technologies using mass spectrometry have enabled the identification of disease-associated volatile organic compound (VOC) metabolites [7–10]. However, the electronic nose (E-Nose) is a portable, simple, rapid, and inexpensive diagnostic device that can differentiate between complex mixtures of disease-associated VOC metabolites without identifying specific chemical species [9–13]. It uses an array of non-specific cross-reactive chemical sensors to record discrete patterns in response to various VOCs in the headspace above a biological sample, producing a unique “fingerprint” by a recognition system [14]. Earlier studies showed that the E-Nose could successfully discriminate between normal and infected urine with bacterial pathogens incubated in a volatile-generating test tube system for 4–5 h based on VOC patterns [15].

The purpose of this research was to assess the viability of the E-Nose technology as a rapid system in clinical practice for the detection of BSIs caused by two commonly isolated Gram-negative bacterial species: *E. coli* and *K. pneumonia*.

## Materials and methods

### Materials

One hundred and five blood samples were randomly collected from patients referred to the Microbiology Department, Medical Research Institute, Alexandria University, Alexandria, Egypt, as they arrived for blood culture examinations from June to December 2021. Blood samples were collected in BD BACTEC™ 442260 blood culture bottles (Standard/10 Aerobic/F culture vials, Devine Medical). Of these, 30 BACTEC blood culture bottles tested positive for *E. coli*, another 30 were positive for *K. pneumonia*, while the remaining 40 tested negative for bacterial infections and served as the control group. Other Gram-positive and Gram-negative bacteria were found in five blood culture bottles, but their prevalence was too low to include them in the analysis.

All human subjects used in this study gave informed consent and the research was conducted following “The Code of Ethics of the World Medical Association (Declaration of Helsinki).” All study participants voluntarily agreed to participate in the study and gave their written informed consent before being enrolled. The study protocol was approved by the Ethics Committee of the Medical Research Institute, Alexandria University, Alexandria, Egypt.

### Methods

#### Bacterial identification and growth conditions

Bacteria were initially identified by streaking them onto blood and MacConkey agar, incubating the plates at 37°C in an aerobic environment, and observing the typical cultural characteristics of the colonies (i.e., size, shape consistency, pigmentation, and hemolysis) [4, 5]. Subsequently, the isolates were subjected to biochemical identification and antimicrobial susceptibility (AST) testing with the VITEK 2 system (bioMérieux, Inc., Durham, NC, USA).

The VITEK 2 compact system employs a 64-well barcoded card labeled with a unique identification number, card type, expiration date, and batch number, to conduct 64 biochemical tests and a turbidimetric approach for 20 antimicrobial agents for susceptibility testing. The VITEK 2 ID-GN card can detect and identify a subset of Gram-negative microorganisms that cannot ferment glucose and 154 different species of Enterobacterales in 10 h. Results from the VITEK 2 AST for the most important aerobic Gram-negative bacilli can be obtained in less than 18 h [16].

#### Electronic nose measurements

Blood culture bottles were incubated for 24 h at 37°C, followed by 2 more hours before commencing measurements at room temperature (25°C), to maximize headspace bacterial VOC yields. All blood samples were measured using a portable E-Nose (PEN3, Airsense Analytics GmbH, Schwerin, Germany) in our laboratories. E-Nose sensors continuously recorded changes in resistance (*R*_*i*_) and relative conductance (*G/G*_*o*_), identifying a distinct VOC composition in the headspace of individual blood culture bottles. Table 1 lists the reference compounds and sensitivity limits for the PEN3 E-Nose with 10 metal-oxide semiconductor sensors, as provided by the manufacturer [10].

**Table 1:** AIRSENSE PEN3 electronic nose (E-Nose) 10 metal-oxide semiconductor sensors and sensitivity characteristics as provided by the manufacturer^11^

Sensor	Name	Reference	Substance
S1	W1C	C_6_H_5_CH_3_, 10 ppm	Aromatic compounds
S2	W5S	NO_2_, 1 ppm	Nitrogen compounds, broad range, very sensitive to negative signals
S3	W3C	C_6_H_6_, 1 ppm	Ammonia, aromatic compounds
S4	W6S	H_2_, 100 ppb	Selective to hydrogen (breath gases)
S5	W5C	CH_3_CH_2_CH_3_, 1 ppm	Alkanes, aromatic compounds, less polar compounds
S6	W1S	CH_3_, 100 ppm	Methane ca. 10 ppm (environment), broad range,
S7	W1W	H_2_S, 1 ppm	Sulfur compounds H_2_S 0.1 ppm, terpenes, and sulfur organic compounds (limonene, pyrazine)
S8	W2S	CO, 100 ppm	Alcohols, partially aromatic compounds, broad range
S9	W2W	H_2_S, 1ppm	Aromatic compounds and sulfur organic compounds
S10	W3S	CH_3_, 10CH_3_, 100 ppm	Selective to methane, high concentrations >100 ppm

**Fig. 1:**
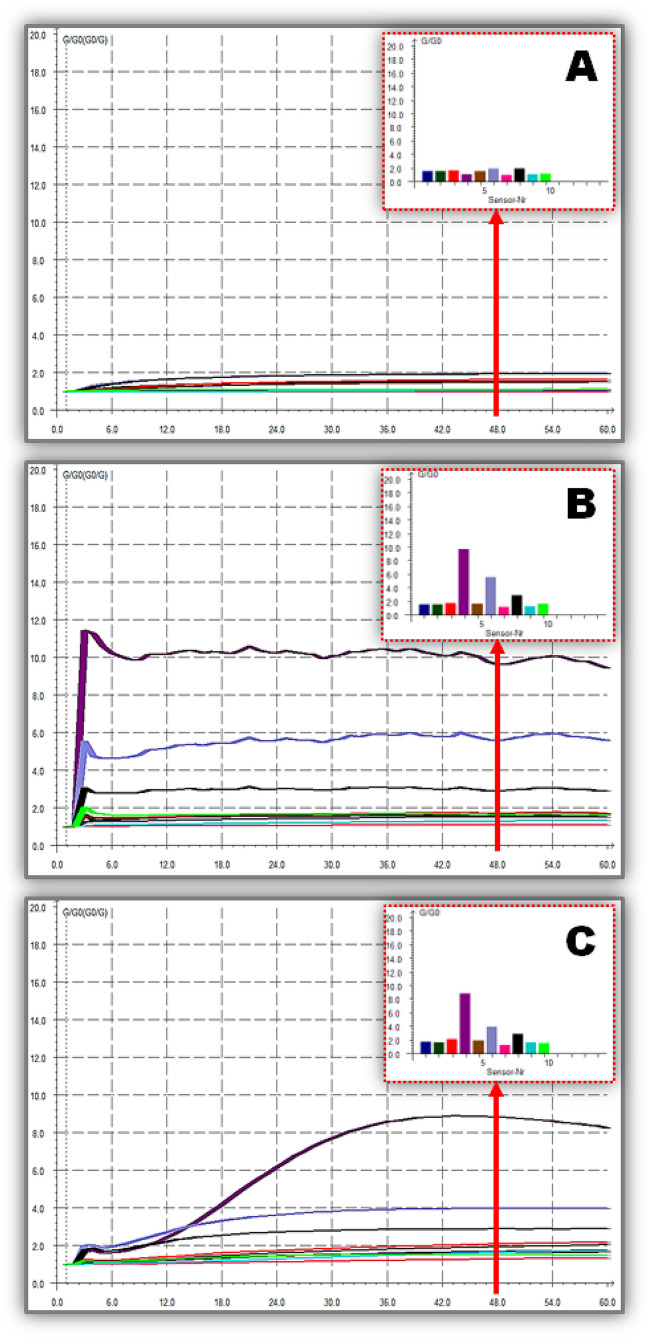
Typical continuous alterations in 10 sensors resistance (*R*_*i*_) and relative conductance (*G*/*G*_*o*_) of an e-nose measurement time-plot for blood culture bottle from **A** a negative control sample for bacterial infections, **B** a positive infected sample with *E. coli*, and **C** a positive infected sample with *K. pneumonia.* In the headspace of blood samples infected with *E. coli* and *K. pneumonia*, sensors S4, S6, and S8, which are sensitive mostly to H_2_, CH_3_, and CO compounds, respectively, were significantly different from negative controls

The E-Nose inlet and the ambient air are connected to each blood culture bottle via Teflon tubing to a long leur-lock needle and a smaller needle perforating the bottle’s seal. Dry air introduces VOCs to the 10-sensor array chamber at a 400 ml/min rate, where solenoid valves switch between measuring the VOCs in the bottle headspace for 60 s and cleaning the sensors with charcoal filter-purified dry air for 50 s. Before connecting another bottle for a new measurement, a 10-s trim time is required to reset the signals to their initial values (*G/G*_*o*_ = 1), as previously described [8–13]. The monitor displays the continuous *R*_*i*_, and *G/G*_*o*_ differential in sensor output patterns for the measurement period, as shown in Fig. 1. Measurement experiments were repeated in triplicates, and sensor array pattern files were saved for further analysis.

#### Statistical analysis

PCA was used to isolate and further evaluate E-Nose 10-sensor patterns in the plateau region at 48 s, where *R*_*i*_ and *G/G*_*o*_ values from 10 to 60 s measurements are steady for any sample in Fig. 1. By eliminating second-order dependencies, PCA reduces the size of a dataset by linearly mapping it onto a smaller number of orthogonal axes [17–19]. Therefore, PCA can determine which principal components best differentiate between *E. coli*, *K. pneumonia*, and negative control samples when projecting sensor patterns from blood culture bottles. For this reason, the largest variance by any projection of the sensor patterns is shown on the *x*-axis as principal component #1 and the second largest variance is shown on the *y*-axis as principal component #2 [8–13].

To graphically represent and quantitatively assess the success of classifying blood culture bottles, we used ellipse-bounded cluster plots of E-Nose sensor projections, where the overall variance equals the sum of principal components #1 and #2. Furthermore, analysis of the area under the receiver operating characteristic curve, sensitivity, and specificity were used to determine the classification accuracy of *E. coli*, *K. pneumonia*, and negative controls.

## Results and discussion

BSIs, the main cause of septicemia and septic shock, account for a large percentage of annual deaths around the world. Patients with active BSIs require a prompt diagnosis to prevent the infection from spreading to other parts of the body. Although they are still regarded as the gold standard, traditional microbiology identification methods are time-consuming and costly; hence, it is vital to develop new tools for a cheap, rapid, and accurate diagnosis of BSIs [20]. This study examined the capabilities of a portable E-Nose to diagnose BSIs and distinguish between *E. coli* and *K. pneumonia* from blood culture bottles as an alternative to traditional biochemical testing and VITEK 2 system rapidly and accurately.

**Fig. 2:**
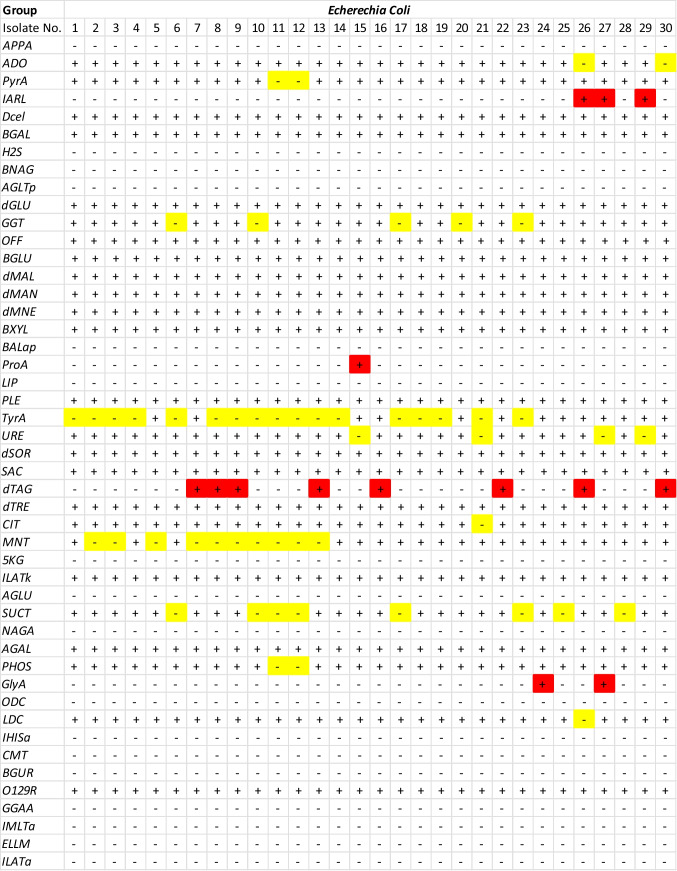
VITEK 2 system results for identifying infected blood culture bottles tested positive for *E. coli*

**Fig. 3:**
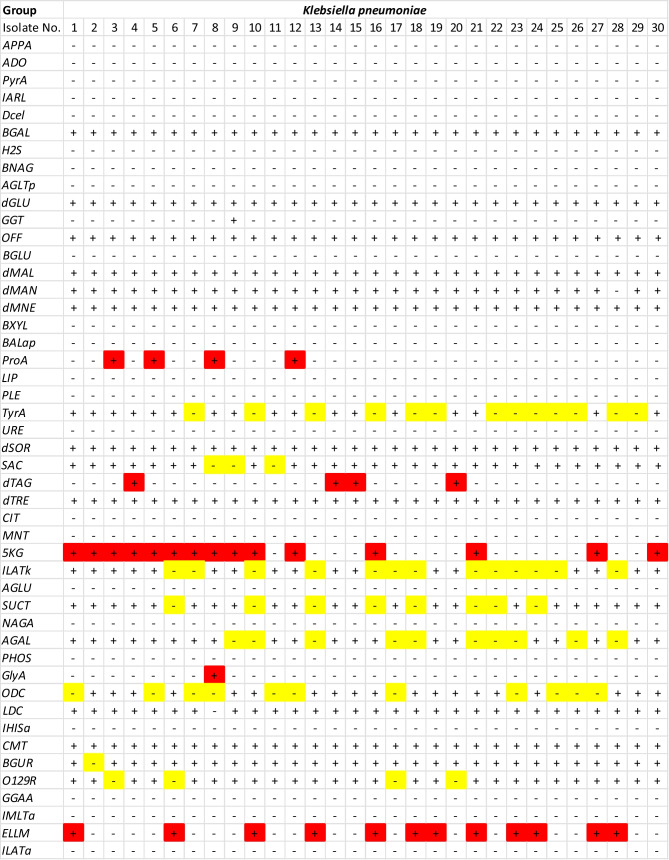
VITEK 2 system results for identifying infected blood culture bottles tested positive for *K. pneumonia*

The VITEK 2 system confirmed infection with *E. coli* and *K. pneumonia* of all positive blood cultures using traditional morphological and biochemical techniques with an overall accuracy of 99%. In accordance with the VITK 2 tests depicted in Fig. 2, all *E. coli* isolates passed the indole, methyl red, and motility tests but failed the Voges-Proskauer, urease, and Simmon citrate utilization tests. In contrast, as shown in Fig. 3, all *K. pneumonia* isolates tested positive for Voges-Proskauer, urease, and Simmon citrate utilization but negative for indole, methyl red, and motility. Since *K. pneumonia* can produce urease enzymes and degrade urea, it tested positive for urease, while *E. coli* did not. These findings are consistent with those of Monteiro et al. [21], who compared the accuracy of the VITEK 2 system to that of conventional PCR and DNA sequencing in the identification of 400 microorganisms, finding an accuracy of 94.7% for the isolates, 100% for the Gram-negative bacilli, and 92.6% for the Gram-positive cocci isolated from blood cultures. However, this and other studies showed that the VITEK 2 system is time-consuming, identifying many species of Enterobacterales and a small subset of Gram-negative microorganisms that cannot ferment glucose within 10–18 h [16]. Consequently, VOC-based techniques for microbial identification coupled with antimicrobial susceptibility testing are likely to yield relevant information for guiding clinical antimicrobial therapy in less than 24 h [22].

Metal-oxide semiconductor sensors in an E-Nose, which rely on gas molecule adsorption to induce a conductivity change quantifying the amount of adsorbed molecules, can be highly responsive to VOCs with molecular weights ranging from 30 to 300 Da [23–25]. In other words, they are sensitive to a wide variety of molecules, including alcohols, esters, ketones, and fatty acids, as well as molecules with sulfur and amine groups, but less so to fully oxidized species (e.g., CO_2_, NO_2_, and H_2_O) [24]. The E-Nose with 10 metal-oxide semiconductor sensors used in this study successfully identified positive blood culture bottles with *E. coli* and *K. pneumonia* from those negative for bacterial infection, as shown in Fig. 1. Relative sensor responses (*G/G*_*o*_) to headspace VOCs of infected blood samples were highly differentiated in a way characteristic to *E. coli* and *K. pneumonia* compared to negative controls. Specifically, sensors S4, S6, and S8, which are sensitive mostly to H_2_, CH_3_, and CO compounds, respectively, were significantly different from negative controls, as supported by earlier research findings.

Changes in VOCs in the headspace of culture bottles inoculated with blood from *E. coli*-infected or non-infected rabbits were documented using automated gas chromatography-ion mobility spectrometry [26]. After 36 h of incubation, the highest achieved total data variance explained by principal components #1 and #2 was only 84.2%, without identification of any chemical compounds. Moreover, methanol (CH_3_OH), ethanol (C_2_H_6_O), and acetone (C_3_H_6_O) were found to be the most common VOCs associated with ATCC 25922 *E. coli* strain in trypticase medium, followed by fatty acid esters such as methyl and ethyl palmitate (C_17_H_34_O_2_ and C_18_H_36_O_2_, respectively) [27]. Furthermore, indole (C_8_H_7_N) was also associated with *E. coli* in trypticase medium due to tryptophan conversion to indole. Likewise, of 365 VOCs associated with the growth of *K. pneumonia* across all media, only 36 were common to all growth media [28]. Ketones (C_n_H_2n_O) were the most abundant compound class in all *K. pneumonia* growth media; however, alcohols, esters, heterocycles, and hydrocarbons were also abundant.

**Fig. 4:**
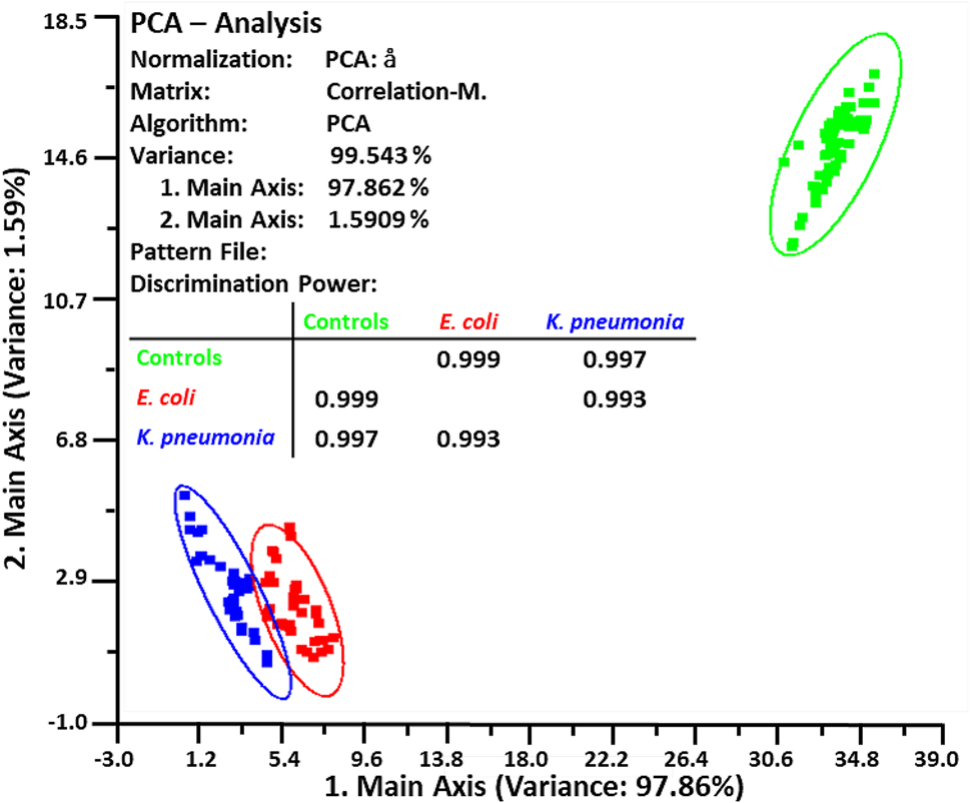
Cluster plot of principal component #1 against principal component #2 for an electronic nose (E-Nose) with an array of 10 metal-oxide sensors applied to the headspace of infected blood culture bottles with *E. coli* and *K. pneumonia* and negative controls

The PCA cluster plot shown in Fig. 4 clearly differentiates between the three groups; the #1 and #2 principal components showed a 97.86 and 1.59% variance, respectively, for a total of 99.45%. AUC values for *E. coli*, *K. pneumonia*, and negative controls were 0.999, 0.997, and 1.00, which signify a 100% accuracy in the diagnosis and identical sensitivity and specificity values of 100% for the three groups. Thus, after 24 h of incubation, the E-Nose correctly identified blood culture bottles from each group, with neither false-positive (negative controls) nor false-negative (*E. coli* and *K. pneumonia*) results. This means that there are substantial differences between the VOC profiles of blood samples taken from patients infected with *E. coli* and *K. pneumonia* and those taken from negative controls. These findings outweigh those of a similar study by Sun et al. [29], who developed an E-Nose made up of 30 metal-oxide and electrochemical gas sensors to detect *E. coli*, *S. aureus*, and *P. aeruginosa* in infected wounds. Without sensor array optimization, they achieved an 86.54% recognition rate using a support vector machine classifier; with optimization using Wilks’ Lambda Statistic and LDA (but not PCA), they were able to get it up to 96.16%. Traditional testing results were in good agreement with those obtained using a combination of direct matrix-assisted laser desorption ionization time-of-flight MS and RT-PCR to identify BSIs down to the genus and species level within about 13.2 h [30].

These results suggest that the E-Nose technology is just as precise as the VITEK 2 and direct matrix-assisted laser desorption ionization time-of-flight MS systems, since it correctly identified BSIs with *E. coli* and *K. pneumonia* in just 120 s. Thus, the E-Nose technology is a practical technique in microbiology laboratories to diagnose BSIs rapidly, precisely, and affordably from blood culture bottles, reducing the high morbidity and mortality rates among hospitalized patients with BSIs.

## Conclusion

The E-Nose technology can be used in microbiology laboratories to diagnose BSIs directly from blood culture bottles, which can be a rapid, accurate, and low-cost alternative to automated blood culture BACTEC and VITEK 2 systems. It can identify the presence of bacterial species like *E. coli* and *K. pneumonia* in the blood culture bottles after a 24-h incubation at 37°C before obtaining a definitive result of the subculture on solid media. It enabled starting a guided empirical antimicrobial therapy on the same day of diagnosis of BSIs, which can be lifesaving for patients with such Gram-negative bacteremia.

In future work, we are planning to expand the study to a larger scale to include a wider variety of bacterial pathogens commonly causing BSI, constructing an online database with the unique and distinct fingerprints to be widely available for microbiologists to identify bacterial pathogens from blood and other clinical specimens. The E-Nose technology, when combined with artificial intelligence capabilities, is a promising technique for use in clinical laboratories to supply presumptive results about the causative agents of infection in various clinical specimens. This information can be critical in making prompt treatment decisions that can save the lives of critically ill patients.

## Data Availability

Metadata used and/or analyzed during the current study will be made available from the corresponding author on reasonable request.
